# Targeting AVIL, a New Cytoskeleton Regulator in Glioblastoma

**DOI:** 10.3390/ijms222413635

**Published:** 2021-12-20

**Authors:** Robert Cornelison, Laine Marrah, Drew Horter, Sarah Lynch, Hui Li

**Affiliations:** 1 Department of Pathology, School of Medicine, University of Virginia, Charlottesville, VA 22908, USA; jrc3hg@virginia.edu (R.C.); lm2ku@virginia.edu (L.M.); dah6zq@virginia.edu (D.H.); sl7hx@virginia.edu (S.L.); 2 Department of Biochemistry and Molecular Genetics, School of Medicine, University of Virginia, Charlottesville, VA 22908, USA

**Keywords:** glioblastoma, AVIL, FOXM1, LIN28B, actin, gelsolin

## Abstract

Glioblastoma (GBM) is the most common adult neural malignancy and the deadliest. The standard of care is optimal, safe, cytoreductive surgery followed by combined radiation therapy and alkylating chemotherapy with temozolomide. Recurrence is common and therapeutic options in the recurrent setting are limited. The dismal prognosis of GBM has led to novel treatments being a serious roadblock in the field, with most new treatments failing to show efficacy. Targeted therapies have shown some success in many cancers, but GBM remains one of the most difficult to treat, especially in recurrence. New chemotherapeutic directions need to be explored, possibly expanding the targeted chemotherapy spectrum in previously unforeseen ways. In this perspective paper, we will explain why AVIL, an actin-binding protein recently found to be overexpressed in GBM and a driving force for GBM, could prove versatile in the fight against cancer. By looking at AVIL and its potential to regulate FOXM1 and LIN28B, we will be able to highlight a way to improve outcomes for GBM patients who normally have very little hope.

## 1. Introduction

**Figure 1:**
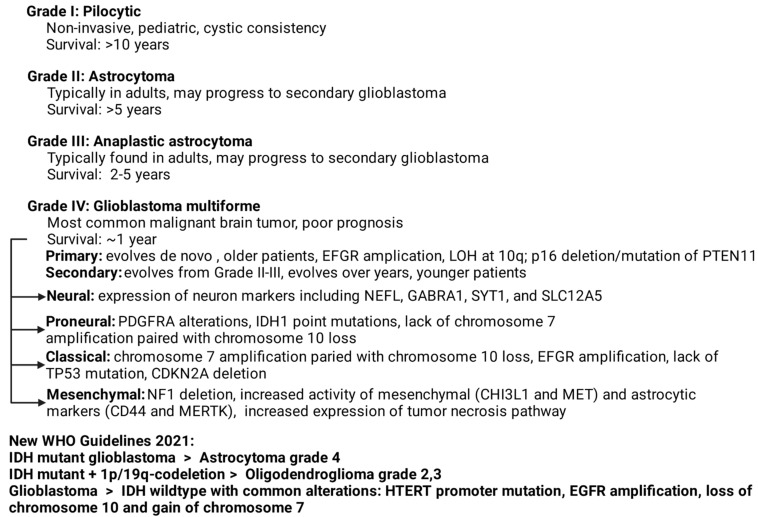
Overall classification of the common forms of astrocytomas. Guidelines from the World Health Organization were updated in 2021. The overall heterogeneity of CNS tumors makes nomenclature and histologic distinction a critical but challenging task in the wake of novel technologies [17,18]. Treatment options at presentation have remained relatively stable for many years with few novel treatments or targeted therapies showing efficacy. The current standard of care is surgery followed by combination radiotherapy and temozolomide chemotherapy in the case of glioblastoma with methylated O^6^-methylguanine-DNA-methyltransferase (MGMT). In cases with unmethylated MGMT, the addition of alternating electric fields therapy has shown promise in clinical trials and is included in front-line treatment in the National Comprehensive Cancer Network (NCCN) guidelines. Due to its tendency to be hypervascularized, the anti-angiogenic bevacizumab is commonly used as a second-line therapeutic in combination with agents like the alkylating mustard carmustine or lomustine [19,20,21]. Resistance to these therapies is based on two major factors: maintenance of stemness and overall dissemination. GBMs rarely metastasize to distant sites, but, through an EMT-like process (glial-to-mesenchymal transition) specific to the brain, they can disseminate widely throughout the CNS, making GBM a disease of the entire brain [22,23,24].

Glioblastoma multiforme (GBM) is the grade IV astrocytoma. As one of the most common and malignant adult neural malignancy, it affects approximately 14,000 people in the United States per year [1,2,3,4]. Astrocytomas are malignancies arising from astrocytes divided into four grades: pilocytic astrocytoma (grade 1, generally non-invasive, pediatric masses), astrocytoma (grade II), anaplastic astrocytoma (grade III), and GBM (grade IV). GBMs are further divided into primary and secondary subtypes based on both biology and differences in overall molecular evolution. The current standard of care is cytoreductive surgery followed by the combination of radiation therapy and alkylating chemotherapy with temozolomide [3,4,5,6,7]. Primary GBMs are thought to arise de novo as the grade IV lesion, and are generally associated with an aging population (age > 50), EGFR amplification, loss of heterozygosity (LOH) at 10q, p16 deletion, and mutation of PTEN [8]. Secondary GBMs evolve from lower grade II–III lesions over several years and are seen in younger patients [9]. Overall life expectancy for primary GBM is only 12–15 months, while lower grade III anaplastic astrocytoma patients survive for 2–5 years [1]. GBM prognosis is so dismal that long-term survivors (LTS) are clinically defined as living >36 months after diagnosis with only 2–5% meeting this criteria [10,11]. The hallmark GBM histologic finding is microvascular proliferation and areas of pseudopalisading necrosis [12,13]. World Health Organization (WHO) classification now separates GBMs into two subgroups by isocitrate dehydrogenase (IDH) gene mutation status, with wild-type IDH accounting for 90% of cases seen [2,14,15]. Molecular categorization clusters GBMs into four subgroups [16], as seen in (Figure 1). 

Cytotoxic chemotherapy has long been a successful, yet problematic treatment for many types of cancer. With each class of chemotherapeutics comes a diverse list of toxicities that make it difficult to distinguish between adverse effects and disease pathology [25]. Antimitotic agents have proven to be very effective in the killing of cancer cells, but by acting on mitosis, lend themselves to causing additional cell death throughout the body. The move to cancer-specific, targeted therapies began with BCR-ABL fusion targeting using imatinib [26]. This opened the cancer field to an entirely new philosophy of therapeutic options in identifying and characterizing differentially expressed genes in cancer as lead targets for pharmaceutical intervention. The initial discoveries of cancer-specific proteins to inhibit led to drugs targeting MEK, b-RAF, ALK, and other major signaling network controllers. However, only 5–7% of novel targeted therapies demonstrate significant efficacy and make it through clinical trials in solid tumors. The overall tumor heterogeneity combined with the complex, redundant signaling networks seen in most solid tumors makes specific, gene-level inhibition unlikely to defeat most advanced cancers.

In reality, the promise of ultra-specific, targeted therapies has yet to be fully realized, especially in aggressive malignancies like GBM. Solid tumors are rapidly evolving entities, and adding specific selection pressures in the form of targeted inhibitors to genetically unstable, rapidly dividing tumors leads to a predictable outcome: chemoresistant, untreatable cancer cells. This has led the targeted therapy field to step back from ultra-specific monotherapies and look for more broad range inhibitors that target entire families of kinases. Imatinib, while being designed to target the cancer-specific BCR-ABL fusion, has been shown to also target other kinases in cancer [22], making it a promising treatment for use outside of cancers with the Philadelphia chromosome. This underscores the importance of moving away from monotherapies and using well-thought-out, outcome-directed combination therapies meant to enhance specific targeting of a pathway or block therapy bypass mechanisms.

Combination therapies are critical for targeted inhibitor success in many cases, as using dual inhibition of the same pathway can lead to synergistic effects. Inhibiting activating mutations of RAF kinases has shown promise in susceptible melanoma patients, but combining RAF inhibition with MEK inhibitors can lead to better outcomes [27,28,29,30]. These combined treatments unfortunately also lead to severe side effects and, in many cases, toxicities requiring termination of treatment. Instead of targeting specific families of enzymes or other “druggable” proteins, there has always been the prospect of identifying cancer-specific genes that mimic the targets of the original cytotoxic chemotherapies, such as tubulins and other cytoskeletal proteins. In this way, they retain their increased specificity to cancer, but by targeting critical cell infrastructure can induce widespread damage to multiple intracellular systems simultaneously.

The idea of identifying a cancer-specific cytoskeletal protein is not new. Some of our strongest cytotoxic agents target the cytoskeleton and inhibit mitosis. The action being primarily on dividing cells gives some cancer specificity in that at least they target rapidly dividing cells. The downside of the approach has been simply that the body has many rapidly dividing cell types, leading to many deleterious toxicities. Chemotherapies targeting the cytoskeleton began in the 1960s with the use of vincristine, a vinca alkaloid, targeting tubulin and disrupting polymerization. Targeting tubulin as a means of preferentially attacking rapidly dividing malignant cells has dominated cytoskeletal agents with the most commonly used being the vinca alkaloids, the taxane stabilizing family (paclitaxel and docetaxel), and the newer epothilones [31,32,33]. Epothilones have come to the forefront of research by demonstrating less toxicity and more specificity, but overall side effects remain high in cytoskeletal targeting. Importantly, disrupting tubulin specifically is catastrophic to all dividing cells, whether malignant or not. A differentially expressed, non-tubulin cytoskeletal protein that could be targeted with a reasonable therapeutic index has been a difficult find. Actin, on the other hand, is required for many malignant phenotypes including metastatic spread, cytokinesis, and cell motility. Recently, our lab discovered a previously seen but relatively uncharacterized oncogenic actin-binding protein, advillin (AVIL), to be upregulated in GBMs as well as other cancers [34]. It is rarely expressed outside of embryonic stages, and in GBM appears to be critical for maintaining tumorigenicity. Advillin may be a relatively cancer-specific cytoskeletal protein that sits at the crossroads of the two therapeutic philosophies: broad range cytotoxic agents that target machinery required for division and a cancer-specific targeted therapy that limits toxicities in normal cells. 

In this manuscript we will give a brief overview of AVIL and its functions, a summary of the known family members and their proto-oncogenic roles, roles of downstream targets of AVIL and their specific functions in GBM, and look at AVIL functions specifically involved in GBM dissemination and immune system evasion. Finally, we will speculate the therapeutic potential of targeting AVIL in GBM. 

## 2. Advillin Overview

Advillin, encoded by the AVIL gene, is one of the members of the gelsolin superfamily of calcium-dependent, actin-binding proteins [35,36]. Actin-binding proteins remodel actin in several different ways and act as modifying components of the overall cytoskeletal network. Advillin can nucleate filaments and promote polymerization, bundle actin to create cytoskeletal networks, disassemble actin through severing and depolymerization, and cap actin at either the pointed or barbed end [36,37]. The role of actin-binding proteins in cancer has been under substantial investigation. The actin cytoskeleton is responsible for a broad range of cellular phenotypes such as maintenance of polarity, movement, invasion, endocytosis, cell division, and trafficking [38]. For cancers to proliferate and invade, the actin-binding proteins are essential for these functions by how they polymerize, depolymerize, and bundle actin using intracellular calcium, and a host of chaperone proteins depend upon the localization and essential function. Advillin appears to be a critical actin-binding protein involved in bundling actin in response to stress and movement [39]. 

**Figure 2:**
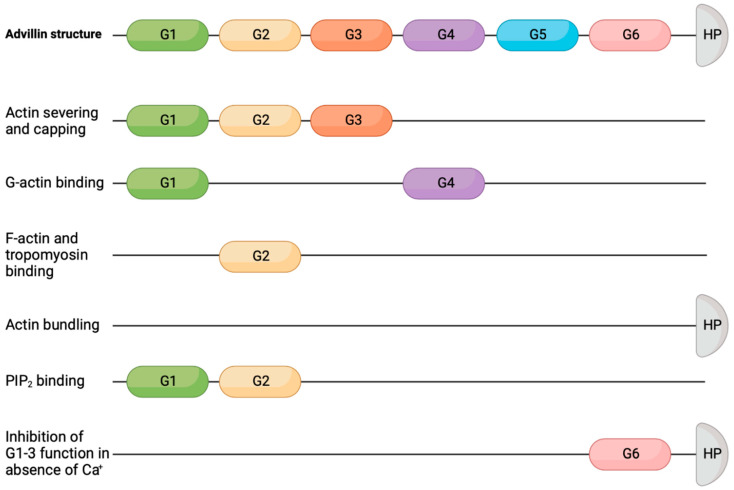
Advillin protein structure and domain functions.

Advillin was identified in an adult murine brain cDNA screen to share 65–75% homology to villin, gelsolin, and adseverin [35]. Similar to other gelsolin superfamily members, Advillin contains six domain structures, termed gelsolin-like (G)1–6, with a carboxy-terminus headpiece domain [40], as shown in (Figure 2). The G1 and G4 domains have been found to bind actin monomers, while G2 binds filaments. The headpiece also contains F-actin binding activity, which has been shown to be involved in actin bundling [41]. Gelsolin contains the G1–6 but does not have the headpiece domain. The headpiece domain seen in advillin is shared with villin (VIL1), villin-like (VILL), flightless 1, and supervillin [40]. 

AVIL is heavily overexpressed in almost 100% of GBMs, while the gene products are virtually non-existent in normal brain tissue [34]. This overexpression was also confirmed via immunohistochemistry assays that were performed on normal brain and glioblastoma tissues. The gene is essential for the proliferation and migration of GBMs [34]. The siRNA-mediated silencing of AVIL resulted in a reduction of cell motility measured by wound healing and live cell imaging, and eventually killed cancer cells expressing AVIL, while the same siRNAs on astrocytes had no obvious effect. In contrast, overexpressing AVIL enhanced cell proliferation and migration and transformed astrocytes *in vivo*. Clinically, higher AVIL expression is associated with worse patient outcomes in GBM as well as lower grade gliomas [34]. A summary of evidence supporting AVIL as a lead target in GBM is shown in Table 1. 

While the exact regulation mechanisms of the advillin are still under investigation, it and similar proteins from its family could be intricately involved in the formation of our most deadly cancers. With its versatile function, it could be hypothesized that AVIL can carry out the functions of other gelsolin/villin family proteins in tumor cells.

## 3. Advillin Family Members and Their Association with Cancer

With important functions in cell motility, ECM interactions, and stress responses, it is unsurprising that gelsolin/villin superfamily members have been found to play roles in tumorigenesis. Gelsolin has been found to regulate EMT in HPV cells, predict patient prognosis in squamous cell carcinoma of the larynx, and suppress apoptotic machinery in neuroendocrine prostate cancer [42,43]. Adseverin expression has been reported to be a marker for cisplatin resistance in bladder cancer and correlate with prognosis in colorectal cancer [44,45]. Calcium signaling is disrupted in GBM, and may provide an abundant source for gelsolin family member activation [46]. PIP2 negatively regulates gelsolin family members, and it is well known that PI3K upregulation in cancer phosphorylates PIP2 to PIP3. This dysregulated signaling may lead to disrupted advillin function in cancer, allowing for promotion and metastasis of GBM.

Glioblastoma is characterized by both resistance to apoptosis and an invasive phenotype. In normal cells, the balance between the pro- and anti-apoptotic machinery regulates mitochondrial membrane permeability, with loss of integrity leading to mitochondrial-dependent cell death. Cellular insults mediated through DNA damage, hypoxia, reactive oxygen species, and nutrient deprivation push this balance to a threshold that results in release of APAF-1, AIF, and cytochrome c from the mitochondria into the cytosol, activating the classical apoptosomes, leading to caspase-dependent death. In cancer, the anti-apoptotic signals raise the threshold of stress and damage required to die and lead to survival of malignant cells. Gelsolin has been found to inhibit apoptosis through multiple mechanisms, including direct interactions with the mitochondria. It interacts with mitochondrial gatekeeper VDAC1, stabilizing the mitochondria and preventing loss of mitochondrial membrane integrity [47]. In this way it can act as common proto-oncogenic BH3 family of anti-apoptotic proteins raising the threshold required for cell death to occur. Glioblastomas also have constitutive caspase-3 activity that promotes cell motility without inducing apoptosis [48]. Gelsolin is an established substrate of caspase-3, producing a cleaved c-terminal fragment that was shown to promote invasion, migration, and metastasis of melanoma cells. Further, the caspase-3 cleaved-gelsolin fragment shows higher efficiency at cleaving F-actin, influencing migration potential [48]. For AVIL specifically, after intracranial xenografts in tumors where AVIL was knocked out by shRNA, there were significantly more apoptotic cells than in the control group [34]. Adseverin, an actin capping and severing protein of the gelsolin family, has also been found to protect against apoptosis-mediated cell death [49]. This indicates that loss of AVIL function inhibits the tumor’s ability to escape some tumor-suppressing mechanisms via apoptosis. 

It has also been shown that gelsolin is key in the inhibition of angiogenesis. To grow and metastasize, tumor cells need a large number of sustained nutrients, oxygen, and the ability to dispose of waste [46]. They can obtain this by inducing the formation of new blood vessels that feed directly to the tumor (angiogenesis), and it has been shown that, in the absence of angiogenesis, the generation of lethal tumor mass is not possible [47]. Endothelial cells with gelsolin knocked out showed significantly higher levels of migration, and when gelsolin was overexpressed, the inhibition of angiogenesis was restored [48]. Furthermore, vascular endothelial growth factor (VEGF) is the primary signal that recruits endothelial cells to migrate [49], and the actin cytoskeleton of endothelial cells is inherently involved in the response to VEGF, which dictates the route for the vascularization of the tumor. AVIL, on the other hand, induces larger, more deadly tumors when overexpressed [45], and thus, angiogenesis is most likely induced by extensive nucleation and growth of actin filaments facilitated by AVIL. 

**Figure 3:**
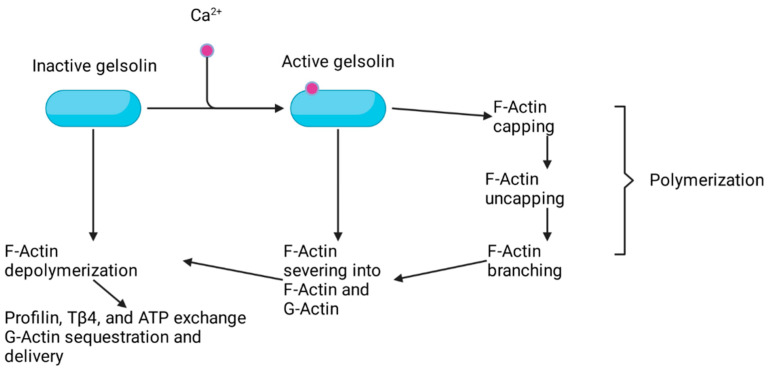
Overview of gelsolin activation through calcium binding and roles in modulating actin structural changes.

Gelsolin has also been found to be packaged into exosomes and secreted, termed plasma gelsolin (pGSN), with this form inhibiting CD8+-mediated tumor immune surveillance [50,51,52]. In this context, secreted gelsolin has been shown to inhibit neoantigen presentation to conventional dendritic cells in the tumor microenvironment, leading to immune evasion with a subsequent increase in glutathione (GSH) expression. Increases in GSH in ovarian cancer increase resistance to platinum chemotherapy [51]. The implication that autocrine signaling through exosomal gelsolin secretion leads to multiple oncogenic phenotypes underscores the breadth of functional roles the gelsolin superfamily of actin-binding proteins can play. A summary of the characterized gelsolin family members’ functional roles on actin is seen in (Figure 3).

Another gelsolin family member, macrophage-capping protein (CapG), is proposed to be a diagnostic biomarker, indicator of prognosis, and predictor of response to treatment across numerous cancer types [53]. High levels of CapG expression have been found to correlate with lymph node metastasis and advanced TNM stage in various cancer types, including glioma [10,53,54,55]. CapG caps the barbed end of actin and inhibits polymerization. Notably, though, growing evidence has supported CapG having non-canonical tumor-promoting activity. In glioma cell lines U251 and U87, overexpression of CapG promoted proliferation while CapG knock-down inhibited cell cycle progression and resulted in an increase of G0/G1 cell cycle arrest and a decrease in cells in G phase [55]. CapG expression also promoted cell migration and invasiveness in glioma cell lines [55]. CapG localizes to both the cytoplasm and nucleus, where it plays a role in invasive phenotypes. Nuclear CapG was found to bind to the epigenetic regulator arginine methyltransferase PRMT5 [56]. PRMT5 methylates histones and is an epigenetic repressor, and PRMT5 inhibition has antitumor effects in glioblastoma models. Gelsolin has been found to be upregulated following IL-6 induction of neuroendocrine transdifferentiation in prostate cancer cells [43] and also to be a coactivator for nuclear receptors including STAT3 and androgen receptor [43,57]. 

## 4. Downstream Targets of AVIL: FOXM1

FOXM1 is a transcription factor belonging to the fox family of proteins unified by its conserved DNA-binding domain known as the forkhead box [58]. Despite a common domain, Fox proteins exhibit a wide range of regulatory activity [59]. FOXM1 regulates several cyclins and kinases and is key to appropriate progression through the cell cycle and mitosis, and the cells of mice without the Foxm1 gene fail to enter mitosis [59]. This transcription factor is mostly expressed in fetal tissue [60] and is typically not expressed in fully differentiated cells [58]. The regulation of FOXM1 is achieved predominantly through phosphorylation, which increases transcriptional activity, but post-transcriptional modification is involved as well [59]. Furthermore, its upregulation has been found to correlate to a variety of cancers including GBM [58]; as such, increased FOXM1 activity could promote cell cycle progression beyond what is appropriate and lead to the formation of tumors [59]. 

The FOXM1 gene contains nine exons and ultimately translates into two alternatively spliced isoforms called FOXM1A and FOXM1B [60]. FOXM1B is the only isoform found to be expressed in glioma cells, implying that this is the isoform relevant to gliomas [60]. FOXM1B, when transfected into mice, caused them to develop aggressive tumors. Inhibiting FOXM1B expression in other mice via siRNA led to no brain tumors and significantly increased survival, implying that FOXM1B has tumorigenic properties [60]. 

Additionally, a correlation has been established between FOXM1 levels and the grade of astrocytoma, with GBM consistently exhibiting the highest levels of the transcription factor while normal brain tissue does not express it [58,60]. An absence of FOXM1 correlates to significantly longer survival in GBM patients, all of which indicates a potential connection between FOXM1 and tumor progression [58]. FOXM1 binds to the promoter of VEGF, the primary growth factor involved in angiogenesis that is frequently overexpressed in glioma cell lines [58]. The absence of FOXM1 corresponds to lowered VEGF *in vitro*, and, in mouse experiments, glioma cells with FOXM1 inhibited were found to have lower VEGF than their counterparts [58]. As such, the interaction between FOXM1 and VEGF is key in driving angiogenesis in glioma cells, which is an important factor in tumorigenesis.

FOXM1 has been linked to EMT in various tumors. It activates MMP-2, which is an enzyme critical to breaking down the extracellular matrix and allowing tumor penetration into the brain. Moreover, MMP-2 is involved in mesenchymal phenotypes in GBM, and increased MMP-2 directly correlates to increased EMT [58]. FOXM1 is, therefore, implicated in the process of tumor invasion, which is a hallmark of GBM as well [58]. 

When AVIL expression is altered, many of the genes with inversely affected expression are targets of FOXM1. More directly, AVIL affects levels of FOXM1 protein but does not affect levels of its mRNA, implying a post-transcriptional regulation [34]. Additionally, there is a direct correlation to half-life, where silencing AVIL reduces FOXM1 half-life and overexpressing AVIL extends FOXM1 half-life [34]. It is postulated that this could be because disrupting F-actin dynamics via silencing AVIL decreases FOXM1 stability [34]. As a result, it has been suggested that FOXM1 is downstream of AVIL [34], and the interaction between the two has potential for elucidating the link between AVIL and tumorigenesis. 

## 5. FOXM1 Regulation of LIN28B and Its Role in GBM

**Figure 4:**
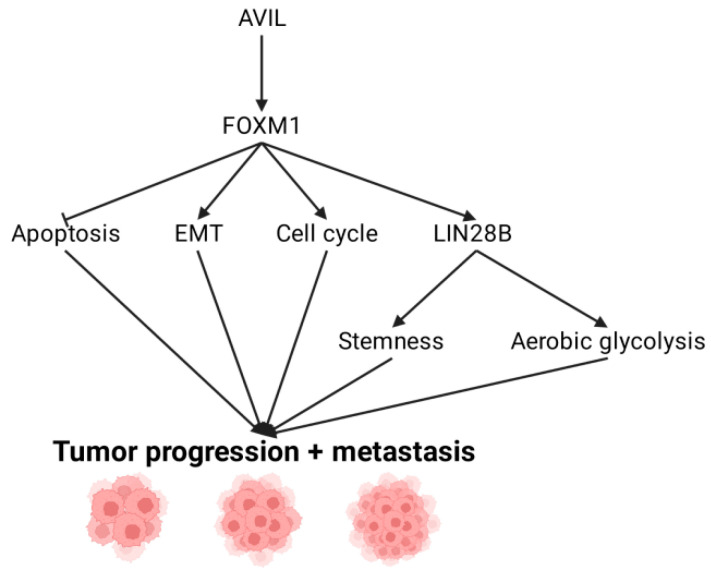
Overview of AVIL oncogenic functions through the FOXM1-LIN28 axis.

LIN28 is an RNA-binding protein associated with oncogenic phenotypes. Higher LIN28 expression has been found to correlate with poorer survival in GBM patients [61]. LIN28 is known to interact with the let-7 family of microRNAs that typically function to suppress tumors, but often have reduced expression in cancer cells [62]. There are two homologs of LIN28, LIN28A and LIN28B, both of which can be expressed in glioma cells [63]. Notably, silencing either AVIL or FOXM1 reduces LIN28B expression; likewise, overexpressing AVIL or FOXM1 increases LIN28B expression. Additionally, expressing LIN28B in cells with silenced AVIL led to some rescuing of tumorigenic effects including proliferation and migration. AVIL has also been found to inversely correlate with let-7 levels [34]. This indicates that LIN28B is likely downstream of both AVIL and FOXM1 [34]. The hypothesized pro-tumor roles of AVIL specifically through the FOXM1/LIN28B axis is seen in (Figure 4). 

LIN28B is overexpressed in cancer cell lines and its overexpression correlates with decreased let-7 and therefore, increased expression of let-7’s oncogenic targets [62]. This occurs because LIN28B blocks let-7 precursors from processing to become mature RNA, therefore inhibiting the production of let-7 miRNAs [62]. Furthermore, LIN28 may play a role in cell cycle progression in glioma cells, which could lead to increased cell proliferation [61]. Overall, it is most likely involved in oncogenic pathways via its interactions with let-7 miRNAs, AVIL, and FOXM1.

Another connection between FOXM1 and LIN28 is that FOXM1 also regulates LINC01094, which acts as a miRNA sponge for MIR-577, a well-characterized tumor suppressor that controls LIN28, as well as b-catenin/wnt signaling and EMT [64,65]. The LINC01094/MIR-577 axis has been shown to control pancreatic cancer progression and metastasis through activation of AKT [64]. It has also been shown to confer radioresistance in renal cell carcinoma specifically through a feedback loop including CHEK2 and FOXM1 [65]. 

## 6. Activating Invasion and Metastasis through EMT

In order to migrate and metastasize, cancer cells take advantage of the epithelial–mesenchymal transition, also called the glial-to-mesenchymal transition (EMT), which is a normal cellular event. During this transition, the cells change their cell-to-cell contacts and are able to move through the extracellular matrix to invade new tissues as a group [66]. It appears that the actin cytoskeleton is heavily involved in the entire transition [67], but its influence is most easily seen in the formation of filipodium and invadopodium. These organelles are vital for the intrusion into new tissues and drive the movement of the tumor cells. The actin-rich structures degrade the extracellular matrix, which allows the tumor to enter new tissues and/or the bloodstream [67].

EMT-like phenotypes in progressing tumor cells have been well documented [68]. In the case of GBM, it is important to make the distinction between EMT and EMT-like phenotypes because EMT indicates that the tumor is epithelial in origin, which is not true of GBM [68]. Our lab found that when silencing AVIL in A172 GBM cells (IDH wild-type/P53 wildtype), there was a dramatic reduction in cell movement in a wound-healing assay [34]. This result was confirmed with live-cell imaging after AVIL knockout. This could indicate that the loss of AVIL function can interrupt the tumor’s ability to invade neighboring brain tissue. Furthermore, AVIL and other villin family proteins have shown to be closely related and colocalized to Arp 3. Additionally, in kidney podocytes, AVIL was found to be integrated with PLCE1, F-actin, and the ARP complex, especially in lamellipodia [39]. Interference with this complex could inhibit the formation of lamellipodia and limit cancer’s ability to spread. 

## 7. Escaping Immune Surveillance 

Natural killer (NK) cells are part of the innate immune system and can coordinate the spontaneous killing of tumor cells [69]. In breast cancer, the involvement of the actin cytoskeleton plays a role in the resistance of tumor cells against NK cells. After NK cells were introduced to a breast adenocarcinoma cell line, there was a large buildup of actin at the contact point that protected the cells from NK-mediated lysis [70]. Furthermore, the inhibition of the Arp 2/3 complex reduced the ability of the tumor cells to form the rapid actin remodeling and made the tumor cells more susceptible to NK lysis by 50–150% [70]. Because AVIL is involved in the formation of dynamic structures such as lamellipodia and filopodia, it is logical to think that AVIL could also be involved with the Arp 2/3 actin assembly complex in the rapid actin response to NK cells. This shows yet another way that an AVIL-based treatment could be effective.

## 8. Discussion

Glioblastoma remains one of the most difficult malignancies to treat with few recent advances seen as truly changing outcomes. The overall complexity of dealing with a primary malignancy of the brain combined with the insidious spread of tumor-initiating cells away from the main lesion makes the development of novel therapeutics a major roadblock in the field. While AVIL may appear superficially to be just another targeted therapy, its role as a major cytoskeletal protein may hold the key to a new generation of targeted therapies that disrupt critical cell infrastructure. Targeting a differentially expressed, cytoskeletal protein may hold promise in keeping toxicity to a minimum while still achieving inhibition across many systems at once. AVIL’s overexpression in tumor cells and lack of expression in noncancerous tissue is promising by itself [34]. It becomes an even stronger candidate when discussing its involvement in known cancer pathways and mechanisms as well as that of the gelsolin superfamily. AVIL’s close relation to the protein gelsolin gives a mechanism by which its knockout could be a death sentence for GBM cells. 

Downstream of AVIL, FOXM1 is implicated in angiogenesis, tumor invasion, and EMT [58], while LIN28B allows expression of oncogenes by inhibiting let-7 miRNAs [62]. The evidence of AVIL acting upstream of both FOXM1 and LIN28B indicates that it likely plays a role in tumorigenesis and proliferation [34]. Additionally, AVIL’s direct role in actin filament modulation provides other mechanisms by which a treatment can act to kill GBM cells [39]. Actin affects tumor mobility, metastasis, and invasion, and treatments targeting AVIL would be able to target the cytoskeleton without affecting tubulin, thus avoiding some deleterious effects in noncancerous dividing cells throughout the body. The prospect of targeting any critical cellular infrastructure can lead to deleterious toxicities regardless of the initial targeting specificity. Pharmaceutical inhibition of actin-binding proteins may be a difficult undertaking in terms of small molecule inhibitors due to the lack of a traditional active site. The gelsolin family members are also quite conserved and care will have to be taken to target AVIL specifically without inhibiting other critical family members. Targeting the headpiece domain or other regions relatively specific to AVIL will be critical in drug design decisions moving forward. Downstream family members could also make specific targeting of the system a reality, but the potential of utilizing AVIL as a means of hitting the entire cytoskeletal system only in cells actively expressing AVIL protein may be the most advantageous considering the malignant potential of GBM cells overall. Modulation of AVIL protein in the cell may also require multiple inhibitors as specific roles of AVIL are more thoroughly elucidated. Rational drug design from atomic-scale starting points may also be required as actin-binding proteins are not commonly included in druggable protein collections. Novel assays for AVIL-specific, oncogenic functional roles in growing tumors will be critical in identifying optimal pharmacologic inhibitors as the field progresses.

As previously mentioned, the metastatic process specific to glioblastoma in terms of cell fate transition is a complex mix of epithelial and mesenchymal gene-specific changes termed glial-to-mesenchymal transition. The players involved include well-known members such as SNAIL and E- and N-cadherin but the interplay between these genes and actin cytoskeletal changes induced by aberrant expression of AVIL still require more research to fully elucidate [71]. Any new gene target associated with such complex pathways involved in GBM tumorigenesis and evolution can lead to a far better understanding of the base mechanisms involved. Our perspective is that AVIL is currently a strong option to move towards the betterment of the standard of care and the outcomes of patients who are inflicted with this horrible disease. 

## Figures and Tables

**Table 1:** Overview of AVIL as a lead target in GBM.

Advillin (AVIL) as a Lead Target in GBM
Expression: AVIL is overexpressed in 100% GBM cell lines and clinical samples, but hardly expressed in astrocyte and normal brain tissues.
Loss-of-function systems: Silencing AVIL caused reduced proliferation and migration of GBM cell culture and xenograft, but had no effect on astrocytes.
Gain-of-function systems: Overexpressing AVIL promoted GBM and astrocyte cell proliferation and migration, and transformed astrocytes.
GBM stem cell/initiating cells: The major therapy resistant cells. GSC/GIC cells express even higher levels of AVIL. Silencing AVIL triggered reduced neurosphere formation and stemness.
Clinical correlation: High AVIL expression is correlated with worse patient survival.
